# Between research and introduction to clinical routine—Experience with niraparib from the compassionate use program in Germany (NOGGO Register Analysis)

**DOI:** 10.1007/s00404-025-08295-x

**Published:** 2026-01-06

**Authors:** Jacek P. Grabowski, Julia Welz, Sabine Heublein, Maja Krajewska, Jolijn D. Boer, Fabian Kraus, Tjadina Arndt, Nicolas Moosmann, Bernhard Heinrich, Tobias Engler, Saida Agabejli, Mustafa Celalettin Ugur, Ralf Witteler, Oliver Albrecht, Harald Müller-Huesmann, Gülten Oskay-Özcelik, Cristina Hettwer, Sabrina Kaiser, Elena Braicu, Jalid Sehouli

**Affiliations:** 1 Department of Gynecology with Center for Oncological Surgery, Charité-Universitätsmedizin Berlin, Berlin, Germany; 2 Nord-Ostdeutsche Gesellschaft Für Gynäkologische Onkologie, Berlin, Germany; 3 Department of Gynecology and Gynecological Oncology, Kliniken Essen-Mitte, Essen, Germany; 4 Department of Obstetrics and Gynecology, University Hospital Heidelberg, Heidelberg, Germany; 5 Department of Gynecology and Obstetrics, University Hospital Ulm, Ulm, Germany; 6 Institute of Biometry and Clinical Epidemiology, Charité University Medicine of Berlin—Corporate Member of Freie Universität Berlin and Humboldt-Universität Zu Berlin, Berlin, Germany; 7 Department of Obstetrics and Gynecology, Ludwig Maximilian University Hospital, Munich, Germany; 8 Department of Medical Oncology, Krankenhaus Barmherzige Brüder Regensburg, Regensburg, Germany; 9 Hematological Oncology Group Practice Dr Bernhard Heinrich and Prof. Dr Markus Bangerter, Augsburg, Germany; 10 Department of Obstetrics and Gynecology, University Hospital of Tübingen, Tübingen, Germany; 11 Department of Gynecology and Obstetrics, Carl Gustav Carus Faculty of Medicine TU Dresden, Dresden, Germany; 12 Agaplesion Diakonieklinikum Hamburg, Hamburg, Germany; 13 Department of Gynecology and Obstetrics, Münster University Hospital, Münster, Germany; 14 Department of Hematology and Internal Oncology, St.Franziskus Hospital Münster, Münster, Germany; 15 Department of Hematology and Oncology, Brüderkrankenhaus St. Josef, Paderborn, Germany; 16 Women’s Cancer Clinic, Berlin, Germany; 17 Clinic for Gynecological Oncology and Operative Gynecology, Bremen, Germany

**Keywords:** PARP-Inhibitor, CUP, Clinical experience, Ovarian cancer, Real-world

## Abstract

**Purpose:**

Ovarian cancer (OC) is frequently diagnosed at a late, advanced stage, resulting in poor survival outcomes. PARP inhibitors like niraparib have shown significant efficacy in high-grade OC, particularly in tumors with homologous recombination deficiency, including BRCA mutations. This study aimed to evaluate dose modifications, safety, tolerability, and the impact on quality of life associated with niraparib in real-world clinical practice.

**Methods:**

This non-interventional, register-based study included patients with platinum-sensitive recurrent OC who received niraparib as part of the compassionate-use program (CUP) in Germany. Clinical baseline characteristics, treatment data, adverse events (AEs), and quality-of-life measures were collected both prospectively and retrospectively across 14 centers. Data analysis was performed using descriptive statistical methods.

**Results:**

Overall, 68 female patients were enrolled in the CUP register. Most patients had good performance status, with no significant comorbidities or concomitant medications. The most frequently reported AEs associated with niraparib were thrombocytopenia, fatigue, and nausea. Approximately half of patients required dose adjustments. AEs were less common in patients with normal physical examination findings, better ECOG performance status, and absence of comorbidities. Prior use of PARP inhibitors or previous treatment-related side effects increased the likelihood of AEs during niraparib therapy. The median treatment duration was 182 days, with disease progression being the most common reason for discontinuation.

**Conclusion:**

Niraparib treatment within the German CUP demonstrated favorable safety and tolerability profiles, supporting its effectiveness in a real-world setting for patients with recurrent OC. These findings are consistent with results from clinical trials, further reinforcing the role of niraparib in this patient population.

**Supplementary Information:**

The online version contains supplementary material available at 10.1007/s00404-025-08295-x.

## What does this study adds to the clinical work

**Table:** 

This manuscript presents real-world evidence supporting the safety and tolerability of niraparib in patients with recurrent ovarian cancer, complementing findings from clinical trials. It identifies key factors associated with adverse event rates and emphasizes the practical importance of dose adjustments in routine clinical practice.	

## Introduction

In Germany, approximately 7000 new cases of ovarian cancer are diagnosed annually. Due to the lack of specific symptoms and limitations of current screening methods, most patients are diagnosed at advanced stages, which significantly impacts survival rates [1]. In FIGO (International Federation of Gynecological Oncology) stages III and IV epithelial ovarian cancer, the 5-year survival rates are approximately 41% and 20%, respectively [2]. The standard treatment for ovarian cancer involves surgery aimed at achieving complete tumor resection, followed by platinum-based chemotherapy and subsequent maintenance therapy. However, approximately 70% of patients with advanced-stage ovarian cancer experience disease recurrence [3].

The historical definition of platinum sensitivity based on the platinum-free interval (PFI) has been replaced by the broader concept of therapy-free interval (TFI). The TFI classification is further subdivided into TFIp (platinum-free interval), TFInp (non-platinum therapy-free interval), and TFIb (biological agent-free interval), reflecting the time elapsed since the last respective treatment. This refined classification aims to better guide treatment decisions in recurrent ovarian cancer [4].

Mutations in the BRCA1 and BRCA2 genes play a significant role in ovarian cancer pathogenesis. These genes are critical for homologous recombination repair (HRR), an essential mechanism for repairing DNA double-strand breaks. Approximately 5–15% of ovarian cancer patients harbor either germline or somatic BRCA1/2 mutations. Among ovarian cancer subtypes, high-grade serous carcinoma is the most common, accounting for 20–25% of cases [5–7]. Patients with BRCA mutations in this subgroup tend to respond better to therapy and have improved clinical outcomes compared to non-BRCA mutation carriers [8–10]. Given the therapeutic and prognostic implications, BRCA testing is crucial in managing ovarian cancer, guiding treatment decisions, and identifying patients who may benefit from targeted therapies such as PARP inhibitors.

Niraparib is a selective inhibitor of poly (ADP-ribose) polymerases 1 and 2 (PARP-1/2) that specifically targets tumor cells by preventing the repair of DNA damage induced by cytostatic agents. The combination of DNA repair inhibition and PARP trapping enhances the therapeutic efficacy of niraparib, making it a potent treatment option for cancers with BRCA mutations or homologous recombination deficiencies (HRD). The safety and efficacy of niraparib as maintenance therapy were evaluated in the Phase 3, randomized, double-blind, placebo-controlled ENGOT-OV16/NOVA trial [9]. This study included patients with platinum-sensitive recurrent ovarian, fallopian tube, or primary peritoneal cancer, predominantly of high-grade serous histology. Niraparib significantly prolonged progression-free survival (PFS) compared to placebo, regardless of BRCA mutation or HRD status. Based on the NOVA trial, niraparib received FDA and EMA approval in 2017 for second-line maintenance therapy [10, 11]. The PRIMA trial, which assessed niraparib as first-line maintenance therapy in patients with advanced ovarian cancer responding to platinum-based chemotherapy, also met its primary endpoint, demonstrating a significant PFS benefit, independent of HRD status [9, 12]. Consequently, niraparib was approved in 2020 for first-line maintenance therapy. However, at the ESMO Congress 2024 (Barcelona, 13–17 September 2024), final overall survival (OS) data from the PRIMA trial (LBA29) were presented after a median follow-up of approximately 6 years. The analysis showed no OS benefit of niraparib over placebo in newly diagnosed advanced ovarian cancer patients at high risk of recurrence. [10].

In 2017, the manufacturer of niraparib launched a Compassionate Use Program (CUP) in Germany to provide patients with early access to niraparib before its official approval. Patients with platinum-sensitive, recurrent high-grade serous epithelial ovarian, fallopian tube, or primary peritoneal cancer who had achieved a response (complete or partial) to platinum-based chemotherapy—regardless of germline BRCA (gBRCA) or HRD status—were eligible for the CUP. Patients were required to have received at least two prior lines of platinum-based chemotherapy. Platinum sensitivity had to be confirmed. The most recent treatment had to be a platinum-containing regimen that resulted in a response assessed by the treating physician as either a complete response (CR) or partial response (PR) [13].

A CUP is a regulatory pathway that allows patients with serious, life-threatening, or chronic diseases to access investigational or unapproved medications outside of clinical trials when no other treatment options are available. These programs are typically intended for patients who have exhausted all approved therapies and do not qualify for ongoing clinical trials. The CUP provides early access to potentially life-saving treatments while ensuring patient safety under regulatory oversight.

This evaluation aimed to assess baseline patient characteristics, including prior therapies, treatment sequencing, BRCA mutation status, patient expectations, and quality of life. Additionally, the analysis focused on efficacy, dosing strategies, toxicity management—with an emphasis on hematological effects—and treatment adherence.

## Methods

### Study setting and participants

This non-interventional register study aimed to document patients receiving niraparib treatment as part of the CUP in Germany. The study did not involve any interventional procedures and sought to recruit up to 192 patients with platinum-sensitive recurrent ovarian cancer who were eligible for PARP inhibitor (PARPi) maintenance therapy across up to 54 sites in Germany. The decision to include patients in the CUP was made exclusively by the treating physician based on established medical practice. Data were collected prospectively and retrospectively, depending on whether patients were already undergoing niraparib therapy, and continued until disease progression or therapy discontinuation for other reasons.

### Objectives

The primary objective of this study was to assess dose adjustments of niraparib, including dose reductions, modification patterns, and reasons for changes. Secondary objectives included evaluating safety, tolerability, and treatment adherence, identifying prognostic risk factors for adverse events, and determining the average dose following adjustments. Additionally, the duration of therapy and its impact on quality of life—measured using the SF-12 questionnaire—were analyzed. Only patients actively receiving niraparib were asked to complete the SF-12 questionnaire.

### Assessments

Before initiating maintenance therapy, baseline data were collected, including demographics, medical history, tumor characteristics (e.g., histology, FIGO stage, residual tumor status), recurrence details, comorbidities, current medications, height, weight (BMI), vital signs, physical examination, ECOG performance status, BRCA status, and blood counts. The SF-12 questionnaire and patient expectations for niraparib were optionally recorded to assess quality of life.

Physicians documented niraparib and subsequent treatments from therapy start until death, withdrawal, or study end, defined as 24 months of follow-up. While there were no fixed time points, follow-up visits were recommended every 3 months according to German S3 guidelines.

During follow-up, data collection included comorbidities, medications, BMI, vital signs, physical examination findings, ECOG status, and lab values. CBCs were required before treatment, weekly from Day 14 for 1 month, monthly for the next 11 months, and afterward as clinically indicated. Additional blood tests could be performed at the physician’s discretion. AEs, SF-12 responses (optional), treatment duration, dose modifications, and reasons for adjustments were documented.

Thirty days after the final niraparib dose, follow-up data were collected again, including AEs and treatment details.

Any incidents of misuse, abuse, overdose, medication errors, accidental/occupational exposure, pregnancy, or lactation were reported as AEs. Events of special interest—such as myelodysplastic syndromes (MDS), acute myeloid leukemia (AML), other secondary malignancies, pneumonitis, and embryo-fetal toxicity—had to be reported within 24 h, following the serious AE reporting protocol.

### Statistical methods

The statistical evaluation of this study was purely descriptive and exploratory, as no predefined hypotheses were established. All patients who received at least one dose of niraparib were included in the analysis. Statistical analyses were performed using R software (R Core Team, Version 4.4.1, 2024).

For categorical variables, summary tables were generated to display absolute and relative frequencies. For continuous variables, descriptive statistics were calculated, including mean, median, standard deviation, minimum, and maximum values. The results of these tests were interpreted descriptively rather than confirmatorily. As a non-interventional study, missing data were anticipated. No statistical imputation methods were used. For categorical and continuous variables, analyses were based solely on valid cases. All data were checked for implausible values. Due to the study’s descriptive nature, outliers were not excluded but were noted where relevant.

To explore potential prognostic risk factors for AEs, Pearson’s correlation coefficient was used for continuous variables, while contingency tables were constructed for categorical variables.

## Results

### Patients

A total of 70 female patients across 14 participating study centers were included in the register. However, due to capacity constraints at one site, two patients could not be documented, resulting in 68 patients being successfully recorded and included in the present analysis.

The median age of participants was 64 years. The majority of patients had no documented comorbidities (54.7%) and were not receiving concomitant medications (62.5%). Among those with comorbid conditions, cardiovascular (17.2%) and endocrine disorders (14.1%) were the most frequently reported (Table 1). Most participants had advanced disease, predominantly classified as stage IIIC (35.4%) or stage IVB (15.4%). Nearly half of patients received supportive therapy for their last chemotherapy, primarily for emesis (69.0%) and infection (51.7%), with common therapies including antiemetics (62.1%) and G-CSF (13.8%). For 64 patients (94.1%) laboratory parameters were documented at baseline. Most blood parameters were within normal ranges, including hemoglobin, erythrocytes, leukocytes, platelets, and serum creatinine, with clinically significant out-of-range values being rare.

**Table 1:** Patient demographics/baseline disease characteristics

	Total *N* = 68
Age, median (years) [min–max]	64 [28–88]
BMI, median (kg/m^2^) [min–max]	24.0 [18.6–40.1]
ECOG PS, *n* (%)	
0 1 Not performed	43 (67.2) 15 (23.4) 6 (9.4)
Comorbidities, *n* (%)	
No comorbidities	35 (54.7)
Cardiovascular disease	11 (17.2)
Gastrointestinal disease	5 (7.8)
Metabolic disorders	9 (14.1)
Musculoskeletal and connective tissue disorders	3 (4.7)
Neurological disease	4 (6.2)
Other malignancies	1 (1.6)
Pulmonary disease	3 (4.7)
Renal disease	2 (3.1)
Other	19 (29.7)
Comedications, *n* (%)	
No concomitant medication used	40 (62.5)
Analgesic	2 (3.1)
Anticoagulant	6 (9.4)
Antidepressants	3 (4.7)
Antidiabetic; Gastric acid inhibitor	5 (7.8)
Antiemetic	1 (1.6)
Antihypertensives	14 (21.9)
Cholesterol lowering drug	3 (4.7)
Dietary supplements	4 (6.2)
Diuretic medication	1 (1.6)
Inhalative bronchodilators	1 (1.6)
Other Comedication	13 (20.3)
Thyroid hormone	7 (10.9)
FIGO, *n* (%)	
I	2 (3.0)
II	6 (9.2)
III	32 (49.2)
IV	18 (27.7)
Not specified	7 (10.8)
Lymph node involvement, *n* (%)	
N1	31 (54.4)
N0/not specified	26 (45.6)
BRCA status, *n* (%)	
Tested	36 (52.9)
BRCA Germline	6 (16.7)
BRCA Somatic	18 (50.0)
Not tested	32 (47.1)
Firstline therapy, *n* (%)	
Primary surgery	66 (98.5)
Chemotherapy	
Carboplatin	58 (92.1)
Gemcitabine	2 (3.2)
PEG-liposomal Doxorubicin	0 (0)
Paclitaxel	2 (3.2)
Other chemotherapy drug/Maintenance therapy	1 (1.6)
PARP-Inhibitor	1 (1.5)
Relapse therapy, *n* (%)	
Chemotherapy	
Carboplatin	58 (90.6)
Gemcitabine	2 (3.1)
Bevacizumab	0 (0)
PEG-liposomal Doxorubicin	2 (3.1)
Paclitaxel	1 (1.6)
Other chemotherapy drug/Maintenance therapy	1 (1.6)
PARP-Inhibitor	1 (1.5%)

*BMI* Body-mass-index, *ECOG PS* Eastern Cooperative Group Performance Status, *FIGO* Federation Internationale de Gynecolgie et d’Obstetrique (in French) International Federation of Gynecology and Obstetrics (in English), *PARP* Poly (ADP-ribose) polymerase

### Treatment duration

The median duration of niraparib therapy was 182 days (approximately 6 months; mean: 331.6 ± 414.0 days), with a range from 9 days to over 6 years. The follow-up period concluded at the end of the study, which was defined as 24 months of follow-up. Tumor progression was the most frequent reason for treatment discontinuation, accounting for 44.1% of cases.

### Dose adjustments

Almost all patients (93.8%) started treatment with a niraparib dose of 300 mg. Only four patients started with 200 mg. Approximately half of the patients (48.4%) did not require any dose adjustments for niraparib. In contrast, 42.2% underwent one dose modification, and a smaller proportion (6.3%) required two or more adjustments. The most common reasons for dose adjustments were thrombocytopenia (52.5%), anemia (27.9%), and fatigue (8.2%). Similarly, treatment interruptions were most frequently attributed to thrombocytopenia (28.8%), followed by anemia (12.1%) and nausea (7.6%). After dose adjustment the average dose level was 243.0 ± 73.9 mg (median: 300 mg; range: 100 mg–300 mg).

### Safety

Most patients (80.9%) had at least one AE during the study period. The most common adverse events observed across all doses of niraparib were thrombocytopenia (35.3%), fatigue (16.5%), and nausea (25.0%). A total of 17 serious adverse events (SAEs) were recorded during the study period. Of these, 14 events (87.5%) were classified as recovered or resolved, one event (6.2%) as recovering or resolving, and one event (6.2%) as not recovered or unresolved. The outcome for one SAE was not documented. The most common serious adverse events observed across all doses of niraparib were thrombocytopenia (4.4%) and anemia (2.9%). None of the SAEs resulted in a fatal outcome (Table 2).

**Table 2:** Adverse events

	Total *N* = 68
Number of patients with at least one AE, *n* (%)	
Total	55 (80.9)
Treatment related	46 (83.6)
CTCAE grade ≥3	22 (40.0)
Hematological toxicities	32 (58.2)
Hematological toxicities grade ≥3	15 (27.3)
Most common adverse events*, *n* (%)	
Thrombocytopenia	
Any grade	24 (35.3)
CTCAE grade ≥3	7 (10.3)
Fatigue	
Any grade	18 (26.5)
CTCAE grade ≥3	1 (1.5)
Nausea	
Any grade	17 (25.0)
CTCAE grade ≥3	1 (1.5)
Anemia	
Any grade	16 (23.5)
CTCAE grade ≥3	7 (10.3)
Constipation	
Any grade CTCAE grade ≥3	11 (16.2) 0 (0.0)
Dyspnea	
Any grade	11 (16.2)
CTCAE grade ≥3	1 (1.5)
Insomnia	
Any grade	11 (16.2)
CTCAE grade ≥3	0 (0.0)
Abdominal pain	
Any grade	7 (10.3)
CTCAE grade ≥ 3	0 (0.0)
Dizziness	
Any grade	7 (10.3)
CTCAE grade ≥ 3	0 (0.0)
Number of patients with at least one SAE, n (%)	
Total	12 (17.6)
Treatment related	5 (7.4)
Fatal	0 (0)

*AE* Adverse event, *SAE* Serious adverse event, *CTCAE* Common Terminology Criteria for Adverse Events

*The most common adverse events were reported by at least 10% of the patients and are listed in order of frequency

### Prognostic risk factors for side effects

Pearson correlation analyses revealed that several baseline factors showed weak to moderate associations with the occurrence of at least one AE during niraparib treatment. In particular, lower baseline sodium levels and platelet counts were more strongly correlated with an increased likelihood of experiencing AEs. In contrast, variables such as age, BMI, and other blood parameters—including hemoglobin, leukocyte, and neutrophil counts—demonstrated only weak or minimal correlations with AE occurrence (see Supplementary Table 1).

Overall, patients with normal physical examination findings and better ECOG performance status experienced fewer AEs. Patients without comorbidities also had a lower incidence of AEs. In first-line therapy, patients who had not previously received PARP inhibitors or those who experienced side effects during prior treatments were more likely to develop AEs when treated with niraparib. A similar pattern was observed in relapse therapy, where patients who had previously received PARP inhibitors or experienced side effects during relapse treatment were also more likely to experience AEs (Table 3)

**Table 3:** Contingency table

	At least one AE with niraparib	No AE
Physical examination at baseline Normal Conspicuities NA	45 (83.3) 5 (9.3) 4 (7.4)	13 (92.9) 0 (0.0) 1 (7.1)
ECOG PS Fully active Restricted in physically strenuous activity but ambulatory Not performed NA	30 (55.6%) 14 (25.9%) 6 (11.1%) 4 (7.4%)	13 (92.9%) 1 (7.1%) 0 (0.0%) 0 (0.0%)
Comorbidities at baseline No comorbidities Cardiovascular disease Neurological disease Renal disease Pulmonary disease Gastrointestinal disease Other malignancies Musculoskeletal and connective tissue disorders Endocrine disorders Other comorbidities NA	23 (42.6) 7 (13.0) 3 (5.6) 1 (1.9) 2 (3.7) 2 (3.7) 1 (1.9) 1 (1.9) 3 (5.6) 7 (13.0) 4 (7.4)	12 (85.7) 1 (7.1) 0 (0.0) 0 (0.0) 0 (0.0) 1 (7.1) 0 (0.0) 0 (0.0) 0 (0.0) 0 (0.0) 0 (0.0)
Side effects in first line therapy Yes No Unknown NA	16 (29.6) 19 (35.2) 17 (31.5) 2 (3.7)	0 (0.0) 10 (71.4) 4 (28.6) 0 (0.0)
Side effects in relapse therapy Yes No Unknown NA	20 (37.0) 21 (38.9) 11 (20.4) 2 (3.7)	2 (14.3) 10 (71.4) 2 (14.3) 0 (0.0)

*ECOG PS* Eastern Cooperative Group Performance Status

### Quality of life

Regarding quality of life, the SF-12 questionnaire was completed by only three patients at baseline, and no subsequent SF-12 data were collected during follow-up visits, including at the end-of-treatment visit. Consequently, a comprehensive assessment of quality of life over time could not be performed due to the insufficient number of data points. At the last documented visit, the ECOG performance status was 0 in 39 patients (69.6%), 1 in 15 patients (26.8%), and 2 in 2 patients (3.6%).

## Discussion

Our observations within the CUP for niraparib showed that, under routine conditions, niraparib was well tolerated by patients with recurrent ovarian cancer. The toxicity profile observed was consistent with the results of the clinical trial program for niraparib maintenance in both primary and recurrent ovarian cancer. Our findings align with the data from the Phase III PRIMA trial, which evaluated the safety and efficacy of niraparib as first-line maintenance therapy in patients with newly diagnosed advanced ovarian cancer who had responded to first-line platinum-based chemotherapy and were at high risk for disease progression or death due to multiple negative prognostic factors [9]. With approximately 5 years of additional follow-up, long-term safety data from the PRIMA trial remained consistent with previously published observations and with the known safety profile of niraparib [9, 14–17]. At the primary analysis, the most frequent severe (CTCAE grade ≥3) treatment-emergent toxicities in the niraparib arm included anemia (31.0%), thrombocytopenia (28.7%), and neutropenia (12.8%). Importantly, there were no treatment-related deaths reported in the study. These findings confirm that hematologic adverse events are the predominant category of severe toxicity associated with first-line niraparib maintenance, consistent with its pharmacologic profile and other trials on PARPi [9].

At the time of the CUP, no upfront reduced starting dose was recommended. In the case of non-hematologic, treatment-related adverse reactions of CTCAE Grade 3 or higher, where prophylactic measures were not considered feasible or the adverse reaction persisted despite appropriate management, treatment with niraparib was to be withheld for a maximum of 28 days or until the adverse reaction had resolved to Grade 1 or lower. Once resolved, niraparib could be resumed at a reduced dose according to the predefined dose modification scheme. A maximum of two dose reductions were permitted. If a CTCAE Grade 3 or higher treatment-related adverse reaction had persisted for more than 28 days while the patient was receiving niraparib at a dose of 100 mg per day, treatment was to be permanently discontinued [13]. Nevertheless, our findings indicated that a few patients had initiated treatment with a 200 mg starting dose of niraparib instead of the recommended 300 mg. More than half of the patients required a dose adjustment for niraparib.

Long-term follow-up confirmed that a weight-adjusted individual starting dose improved the safety profile of niraparib compared to a fixed starting dose [14]. According to the current German product label, the recommended starting dose is 200 mg once daily, or 300 mg for patients weighing ≥77 kg with baseline platelet counts ≥150,000/μl [18]. The observed dose adjustments and therapy duration in our study were in line with findings from previous clinical trials. Berek and colleagues demonstrated that individualized dosing of niraparib reduced overall adverse events, with thrombocytopenia being transient and typically occurring within the first month of treatment. The effectiveness of this dose modification strategy was evidenced by the relatively low number of patients who discontinued due to toxicity [17].

Finally, quality of life is an important factor to consider. As documented in the PRIMA trial, there was a strong association between being progression-free and maintaining health-related quality of life. This observation highlights the long-term clinical benefit of niraparib [10]. No contradictory signals were found in the present analysis. The distribution of ECOG performance status at the last documented visit suggests that niraparib was generally well tolerated over the long term in this patient population.

Compassionate use is a treatment option that allows the use of an unauthorized medicine. Under strict conditions, products in development can be made available to groups of patients with diseases for which no satisfactory authorized therapies exist, and who are unable to participate in clinical trials. Compassionate use programs are coordinated and implemented by individual countries, each of which sets its own rules and procedures [11]. These programs aim to facilitate and improve access to compassionate use, favoring a common approach regarding the conditions of use, distribution, and the targeted patients for the compassionate use of unauthorized new medicines. Such programs are only implemented if the medicine is expected to help patients with life-threatening, long-lasting, or seriously debilitating illnesses, which cannot be adequately treated with any currently authorized medicine. The medicine must be undergoing clinical trials or have entered the marketing authorization application process. While early studies will generally have been completed, the safety profile and dosage guidelines may not be fully established [11].

A key strength of this study is its real-world, multicenter design, which provides valuable insights into the use of niraparib outside the controlled environment of clinical trials. The inclusion of both prospective and retrospective data from 14 centers enhances the generalizability of the findings. Additionally, the focus on dose modifications, safety, and quality of life under routine clinical conditions reflects practical aspects of patient management, offering relevant guidance for clinicians treating recurrent ovarian cancer. The relatively large sample size for a compassionate use setting also adds robustness to the analysis.

However, certain limitations must be acknowledged. As a non-interventional, register-based study, the absence of a control group restricts the ability to draw causal conclusions. The observational nature may also introduce reporting bias or inconsistency in data documentation across centers. Furthermore, the lack of standardized follow-up intervals and reliance on real-world documentation may limit the precision of adverse event reporting and quality-of-life assessments. Finally, the study population may have been subject to selection bias, as patients enrolled in compassionate use programs are typically highly selected and may not fully represent the broader patient population.

The CUP for niraparib enabled the provision of this medication to patients with recurrent ovarian cancer. As a result, many patients in Germany gained access to niraparib and could benefit from its efficacy. Notably, the CUP also enabled the optimization of clinical processes. The earlier a therapeutic concept is integrated into a clinical setting, the sooner it becomes routine. Through a CUP, rapid adaptation to clinical routine is facilitated, allowing innovative therapies to be better integrated into standard practice.

## Authors’ contribution statement

**JP Grabowski**: Protocol development, Data collection, Data analysis, Manuscript writing; **J Welz**: Data collection, Manuscript editing; **S Heublein**: Data collection, Manuscript editing; **M Krajewska**: Data analysis; **J Boer**: Data management, Data analysis, Manuscript editing; **F Kraus**: Data collection, Manuscript editing; **T Arndt**: Data collection, Manuscript editing; **N Moosmann**: Data collection, Manuscript editing; **B Heinrich**: Data collection, Manuscript editing; **T Engler**: Data collection, Manuscript editing; **S Agabejli**: Data collection, Manuscript editing; **MC Ugur**: Data collection, Manuscript editing; **R Witteler**: Data collection, Manuscript editing; **O Albrecht**: Data collection, Manuscript editing; **H Müller-Huesmann**: Data collection, Manuscript editing; **G Oskay-Özcelik**: Data collection, Manuscript editing; **C Hettwer**: Data collection, Manuscript editing; **S Kaiser**: Data collection, Manuscript editing; **E Braicu**: Data collection, Manuscript editing; **J Sehouli**: Protocol development, Data collection, Data analysis, Manuscript editing; All authors read and approved the final manuscript.

## Ethics approval

The study was conducted in accordance with the Declaration of Helsinki, the German Good Clinical Practice (GCP) standards, local laws, regulations, and organizations, with approval obtained from the Ethics Committee of the Charité—Universitätsmedizin Berlin (application number: EA1/149/18), approved on 10.09.2018.

## Consent to participate

Informed consent was obtained from all individual participants included in the study. An exception was made for deceased patients, who could no longer provide consent for participation. In coordination with the ethics committees of the participating study centres, the collection of data from these deceased patients was permissible. This decision was based on the following arguments:

i. The patients had already released their treating physicians from confidentiality obligations in the context of their consent for the Compassionate Use Program.
ii. According to Recital 27 of the General Data Protection Regulation (GDPR), personal data of deceased persons do not fall under the provisions of the GDPR.
iii. A breach of confidentiality according to §203 of the German Criminal Code (StGB) does not occur, as personal data is only transmitted in a pseudonymized form to NOGGO e.V. NOGGO e.V. will never have access to identifying personal data, as only a remote verification of data completeness will take place.
iv. The identification lists used for associating patient numbers will be deleted after the study is completed, thereby ensuring complete anonymization of the data.

## Supplementary Information

Below is the link to the electronic supplementary material.

Supplementary file1 (DOCX 17 KB)

## Data Availability

No datasets were generated or analysed during the current study.
