# Supplementary material for: Between research and introduction to clinical routine—Experience with niraparib from the compassionate use program in Germany (NOGGO Register Analysis)
Source: Arch Gynecol Obstet. 2026 Jan 6;313(1):11. doi: 10.1007/s00404-025-08295-x (PMC12774980; doi:10.1007/s00404-025-08295-x)
Supplement: Supplementary file 1 — Supplementary file1 (DOCX 17 KB) [file 404_2025_8295_MOESM1_ESM.docx]

**Supplemental Material**

| **Supplementary Table 1.** Pearson Correlations |  | | |
| --- | --- | --- | --- |
|  | | At least one AE with niraparib | |
|  |  | Value | n |
| Age | | -0.1483430 | 66 |
| BMI | | 0.1689426 | 26 |
| Hemoglobin at baseline | | 0.1061745 | 63 |
| Erythrocyte at baseline | | 0.0966488 | 58 |
| Leukocyte at baseline | | -0.1422843 | 61 |
| Platelets at baseline | | -0.2517815 | 61 |
| Absolute neutrophil count at baseline | | -0.1092189 | 25 |
| Serum creatinine at baseline | | -0.1070651 | 35 |
| Total bilirubin at baseline | | 0.1156534 | 29 |
| Aspartate aminotransferase at baseline | | 0.0376208 | 23 |
| Sodium at baseline | | -0.3504923 | 22 |
| Potassium at baseline | | -0.0029777 | 24 |
| ECOG PS, Eastern Cooperative Group Performance Status | | | |
